# Whey protein isolate attenuates strength decline after eccentrically-induced muscle damage in healthy individuals

**DOI:** 10.1186/1550-2783-7-30

**Published:** 2010-09-22

**Authors:** Matthew B Cooke, Emma Rybalka, Christos G Stathis, Paul J Cribb, Alan Hayes

**Affiliations:** 1 Exercise Metabolism Unit, Institute for Sport, Exercise and Active Living, School of Biomedical and Health Sciences, Victoria University, Melbourne, Australia; 2 Schools of Medicine and Human Movement Studies, Princess Alexandra Hospital, the University of Queensland, Brisbane, Australia

## Abstract

**Background:**

We examined the effects of short-term consumption of whey protein isolate on muscle proteins and force recovery after eccentrically-induced muscle damage in healthy individuals.

**Methods:**

Seventeen untrained male participants (23 ± 5 yr, 180 ± 6 cm, 80 ± 11 kg) were randomly separated into two supplement groups: i) whey protein isolate (WPH; n = 9); or ii) carbohydrate (CHO; n = 8). Participants consumed 1.5 g/kg.bw/day supplement (~30 g consumed immediately, and then once with breakfast, lunch, in the afternoon and after the evening meal) for a period of 14 days following a unilateral eccentric contraction-based resistance exercise session, consisting of 4 sets of 10 repetitions at 120% of maximum voluntary contraction on the leg press, leg extension and leg flexion exercise machine. Plasma creatine kinase and lactate dehydrogenase (LDH) levels were assessed as blood markers of muscle damage. Muscle strength was examined by voluntary isokinetic knee extension using a Cybex dynamometer. Data were analyzed using repeated measures ANOVA with an alpha of 0.05.

**Results:**

Isometric knee extension strength was significantly higher following WPH supplementation 3 (P < 0.05) and 7 (P < 0.01) days into recovery from exercise-induced muscle damage compared to CHO supplementation. In addition, strong tendencies for higher isokinetic forces (extension and flexion) were observed during the recovery period following WPH supplementation, with knee extension strength being significantly greater (P < 0.05) after 7 days recovery. Plasma LDH levels tended to be lower (P = 0.06) in the WPH supplemented group during recovery.

**Conclusions:**

The major finding of this investigation was that whey protein isolate supplementation attenuated the impairment in isometric and isokinetic muscle forces during recovery from exercise-induced muscle injury.

## Background

Unaccustomed exercise, particularly eccentric exercise in which the muscle lengthens, is the most common method used to elicit muscle damage. Damaged muscle fibers initiate a cascade of reactions that result in a prolonged and complex interaction between protein synthesis and degradation [1]. However, while protein turnover is elevated substantially, degradation usually exceeds synthesis, and thus, protein breakdown results, leading to muscle degeneration and atrophy [2]. These changes in muscle protein ultrastructure normally result in physiological symptoms such as reductions in muscle strength, increased muscle soreness and impaired muscle function [3,4].

Stimulating protein synthesis and minimizing protein breakdown (proteolysis) are the two cellular processes that are essential for muscle recovery after damage [5]. While protein breakdown may be an important process involved in the adaptive response during recovery [6], increasing protein synthetic rates within the muscle during the recovery period is vital for muscle regeneration and hypertrophy. Therefore, strategies that can promote a positive net muscle protein balance during the days following muscle injury are likely to increase the rate of protein synthesis, satellite cell proliferation, but more importantly, enhance the regenerative processes that would benefit athletes and others that perform strenuous/unaccustomed physical activity.

Dietary proteins have an important role in regulating protein metabolism in skeletal muscle [7-9]. Whey protein isolate supplementation has been used effectively to increase muscle size and strength after resistance training [10], with some of these improvements thought to come from improved recovery from the exercise sessions. Compared to regular protein supplements, whey isolate is more effective at increasing blood amino acids and protein synthesis due to its different absorption kinetics and amino acid profile [11]. The high availability of amino acids in whey protein isolate, especially branched chain amino acids (BCAA), is important for protein synthesis in the hours immediately after ingestion. White et al. [12], examined the ingestion of a whey protein after an exercise bout which consisted of 50 maximal isokinetic eccentric quadricep contractions. Muscle strength, muscle soreness and CK were all measured at 6, 24, 48, 72 and 96 hours post exercise, with ingestion of whey protein having no significant effects on these variables implying no change in the rate of muscle recovery. Conversely, Buckley et al., [13] showed whey protein hydrolysate ingestion in the days following an intense exercise bout (100 maximal knee extensions of the knee extensors) improved muscle strength recovery. The authors suggested that the use of partially hydrolysed (pre-digested) form of whey protein isolate may provide quicker delivery of amino acids to the muscle, and ultimately, more rapid recovery of force-generating capacity following muscle injury. The administration of whole proteins in the study by White et al. [12], may explain the lack of improvement in force recovery following damage. Furthermore, only a single dose was given to participants, whereas Buckley et al. [13] continued supplementation following the exercise bout and during the recovery period. It could be suggested that for optimal ergogenic effects and recovery within the muscle, a hydrolysed form of whey protein (or free amino acids) needs to be ingested both immediately following the exercise bout, and in the days during recovery. However, this concept, particularly with eccentric contractions, has not been extensively investigated, as Buckley et al. [13] only followed recovery for 24 hours post-exercise. As such, whether the effects observed were related to muscle damage/regeneration, or simply faster recovery from fatigue, are difficult to determine. Jackman and colleagues [14] supplemented a controlled diet with BCAA and ameliorated the soreness following eccentric exercise. While they did not observe changes in strength measurements, ingestion was on the day of damage and for another 3 days afterwards, rather than for the whole regeneration process.

In our previous study [15], ingestion of creatine monohydrate prior to and following a resistance exercise session indicated a possible attenuation of the amount of damage, and an increase in the rate of functional recovery, compared to a CHO placebo. Similarly, in the current study, given the equivocal data on protein supplementation and muscle recovery, we were interested in establishing whether a commercially available protein supplement can improve recovery from exercise-induced muscle damage, and thus used a CHO placebo as the comparison group. Thus, we supplemented the diet of a group of participants with a hydrolyzed whey protein isolate for 14 days during recovery from an identical resistance training session as used in our previous study [15]. We hypothesized that supplementation with hydrolyzed whey protein isolate will accelerate muscle strength recovery compared to an iso-energetic CHO control after a single bout of eccentric exercise.

## Methods

### Participants

Seventeen healthy, untrained males (23 ± 5 yrs, 180 ± 6 cm, 80 ± 11 kg) volunteered for this study. Descriptive characteristics of the participants are presented in Table 1. Participants fulfilled the inclusion criteria as described in our previous study [15]. Briefly, participants were not allowed to participate in this study if they reported any of the following: 1) participation in a resistance training program; 2) current or past history of anabolic steroid use; 3) any metabolic disorders or taking any thyroid, hyperlipidemic, hypoglycemic, anti-hypertensive, or androgenic medications; 4) ingested any ergogenic levels of creatine, HMB, thermogenics, ribose, pro-hormones (i.e., DHEA, androstendione, etc.) or other purported anabolic or ergogenic nutritional supplements within 6 months prior to beginning the study and to not take any additional nutritional supplement or contraindicated prescription medication during the protocolParticipants agreed not to undertake any physical activity, nor seek any remedy for muscle soreness, other than the supplement provided, for the duration of the study. All participants were informed verbally and in writing, as to the objectives of the experiments, together with the potential associated risks. All participants signed an informed consent document approved by the Human Research Ethics Committee of Victoria University of Australia. All procedures conformed to National Health and Medical Research Council guidelines for the involvement of human participants for research.

**Table 1:** Participant baseline characteristics

Characteristics	CHO	WPH	P-value
Age (yrs)	22 ± 4	24 ± 5	0.13
Weight (kg)	77 ± 14	81 ± 8	0.17
Leg Press 1RM (kgs)	125 ± 51	129 ± 40	0.92
Leg Extension 1RM (kgs)	88 ± 26	84 ± 25	0.70
Leg Flexion 1RM (kgs) Extension	40 ± 8	46 ± 22	0.54

Data are means ± standard deviations of mean. SI unit conversion factor: 1 kg = 2.2 lbs

### Experimental Design

With the exception of the type and timing of the supplement consumed, the experimental design and associated measurements were identical to our previous study [15]. Briefly, 2 weeks prior to the damage session, participants underwent unilateral (dominant limb) concentric 1 repetition maximum (RM) strength assessments as prescribed by the National Strength and Conditioning Association (NSCA) [16], and a familiarisation session of the performance measurements. On the morning of day 1, participants underwent performance measurements - voluntary isokinetic knee flexion and isokinetic/isometric knee extension of each leg using Cybex™ Testing and Rehabilitation System (Cybex International Inc. Ronkonkoma, New York). Strength values were expressed as percentage of pre-exercise values and normalised to contralateral controls as in our [15], and other [17,18], previous studies. A 20-gauge Teflon catheter was placed in a forearm vein, and participants then performed a damage protocol on their dominant leg consisting of leg press, leg extension and leg curls at 120% of the participants' predetermined 1RM for each exercise. The participant completed 40 repetitions (4 sets × 10, with 3 minutes rest between sets) of each exercise at a predetermined cadence (4 seconds), given verbally, which constituted 1 repetition. Participants were given 3 minutes rest between exercises. Blood samples, in order to measure plasma creatine kinase (CK), according to the method of Horder et al. [19], and lactate dehydrogenase (LDH), according to the method of Costill et al. [20], were taken prior to, and then following (30 minutes, 1, 2, and 4 hours), the damage session. Participants returned to undertake the same performance measures and have a further blood sample taken 24 hours post-exercise, and again at the same time at 2, 3, 4, 7, 10 and 14 days following the damage session.

### Dietary Supplementation

Following the resistance exercise session, participants were randomised in a double-blind placebo-controlled fashion into 2 groups: carbohydrate-only (CHO; n = 8) or whey protein-carbohydrate (WPH; n = 9), and issued with their supplement and dosing instructions. The supplements were provided to the participants in identical, unmarked, sealed containers, supplied by AST Sports Science, Golden, Colorado USA. Participants consumed 1.5 grams of either the WPH or CHO control per kilogram of body weight for a period of 14 days. On the testing day, participants ingested their supplement within 30 minutes following resistance exercise session. On every other day, participants would consume this dose in several smaller servings each day, i.e., ~30 g of supplement mixed in water and consumed immediately, once with breakfast, lunch, in the afternoon and after the evening meal following their testing session (i.e. 24, 48, 72, 96 hr and days 7, 10, and 14). The macronutrient content of the supplements was as follows; approx. 90 gms protein, 8 gms iso-energetic carbohydrate, 2 gms fat per 100 gms whey protein supplement (*VP2*™ Hydrolyzed Whey Isolate) and 100 gms iso-energetic carbohydrate per 100 gms of Dextrorotatory Glucose Crystals supplement (DGC™). This dosage is commonly used among resistance-trained athletes to achieve high protein intakes [21]. Therefore, we chose a supplement dose that was characteristic of this population, even though the participants in this study were untrained individuals. Further, AST supplements were made in the USA and underwent independent laboratory testing in the United States for purity and safety. In addition, the content of the supplement was also independently verified (Naturalac Nutrition LTD, Level 2/18 Normanby Rd Mt Eden, New Zealand). Participants were instructed to maintain their typical daily diet throughout the study, with their diet monitored by completion of a written diary as described previously ([22]. During the final recovery week each participant submitted a 7-day written dietary recall for the calculation of macronutrient and energy intake (see Table 2). Participants were also asked to report any adverse events from the supplements in the nutrition diaries provided. No adverse events were reported by the participants.

**Table 2:** Dietary Analyses

	CHO	WPH	P-value
Energy (kcal/kg/day)	30.14 ± 7.3	29.43 ± 5.1	0.85
Protein (g/kg/day)	0.82 ± 0.09	0.85 ± 0.06	0.71
Fat (g/kg/day)	0.94 ± 0.18	0.97 ± 0.18	0.24
Carbohydrate (g/kg/day)	4.58 ± 1.45	4.32 ± 0.95	0.13

Data are means ± standard deviations of mean. SI unit conversion factor: 1 kcal = 4.2 kJ. Values exclude supplementation dose

### Statistical Analysis

Participant characteristics are reported as means ± SD. All other values are reported as means ± SE. Muscle performance data was expressed as a percentage of baseline values, normalized to the contralateral, undamaged limb. Univariate analysis on the CHO group only was used to examine the effects of the damage session on muscle performance variables. Differences between the two groups were analyzed using 2 × 7 (group × time [Day 1, 2, 3, 4, 7 10 and 14) repeated measures analysis of variance (ANOVA) to effectively assess the changes in muscle function/strength following supplementation post-exercise. Blood variables were analyzed using 2 × 14 (group × time [baseline, 30 min, 60 min 2 hours, 4 hours, day 1, 2, 3, 4, 7 10 and 14) repeated measures ANOVA to effectively assess the changes in markers of muscle damage following supplementation post exercise. Least significant difference pairwise comparisons was used to analyze any significant group × time interaction effects. Baseline variables, total work performed during the resistance exercise session and dietary intake between groups were analyzed using a students' t-test. An alpha level of 0.05 was adopted throughout to prevent any Type I statistical errors

## Results

### Participant Characteristics

At baseline there were no differences in the age, body weight or strength level (1RM) between the two groups (see Table 1).

### Total lifting Volume

During the resistance training session, the number of repetitions and weight lifted (120% of 1RM) was recorded for each exercise. Total lifting volume for each group reflects the total number of repetitions multiplied by the total weight lifted performed by each participant for each exercise (see Table 3). No differences were detected between groups.

**Table 3:** Total Lifting Volume

Characteristics	CHO	WPH	P-value
Leg Press 1RM (kg)	18000 ± 7344	18576 ± 5760	0.11
Leg Extension 1RM (kg)	12672 ± 3744	12096 ± 3600	0.49
Leg Flexion 1RM (kg) Extension	5760 ± 1152	6624 ± 3168	0.60

Data are means ± standard deviations of mean. SI unit conversion factor: 1 kg = 2.2 lbs

### Dietary Analysis

One-week dietary analysis (excluding supplementation) revealed no differences in energy, protein, fat and carbohydrate intake between groups throughout the study (see Table 2). Based on supplement dosage of 1.5 g/kg.bw/day, there was no difference in the amount of supplement ingested between the CHO and WPH supplemented groups during the 14-day recovery period.

### Isometric Knee Extension Strength

**Figure 1:**
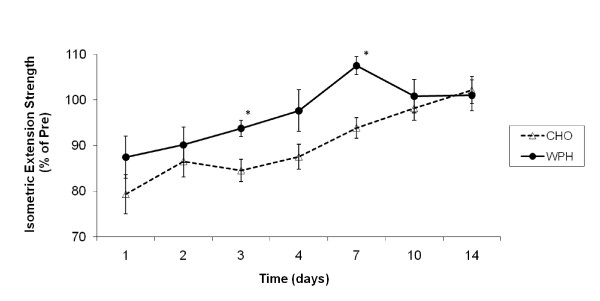
**Effect of CHO and WPH on isometric knee extension muscle strength after exercise-induced muscle damage**. Data (mean ± SE) represents isometric knee extension muscle strength expressed as a percentage of pre-exercise strength taken during the 14 days recovery. * represents (p < 0.05) difference between groups

Pre-exercise absolute values for isometric knee extension strength were 314 ± 27 Nm and 290 ± 17 Nm for CHO- and WPH-supplemented groups, respectively, and were not significantly different. Univariate analysis revealed a significant main effect for time [F(8,104) = 16.750, P < 0.001, effect size(η^2^) = 0.563] and group [F(1,13) = 5.402, P = 0.037, effect size(η^2^) = 0.294]. Reductions in strength (expressed as a percentage of pre-exercise strength) persisted for 7 days and were approximately 21% lower 24 hours post-exercise (P < 0.001), 14% lower 48 hours after (P < 0.01), 16% lower 72 hours into recovery (P < 0.01), 13% lower 96 hours after (P = 0.03), and 7% lower day 7 into recovery (Figure 1). Reductions in strength (significant up to 96 hours post-exercise) were also observed in the WPH supplemented group, albeit smaller reductions than in the CHO group. As such, a significant group by time interaction was group was observed [F(8,104) = 1.854, P = 0.039, effect size(η^2^) = 0.125], with subsequent post-hoc analysis revealing higher isometric knee strength in the WPH group compared to the CHO group 3 days (P = 0.03) and 7 days (P = 0.009) following the resistance exercise session (Figure 1), with a strong tendency also at 4 days (P < 0.08).

### Isokinetic Knee Strength

**Figure 2:**
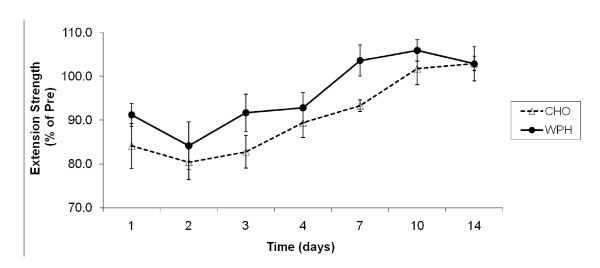
**Effect of CHO and WPH on isokinetic knee extension muscle strength after exercise-induced muscle damage**. Data (mean ± SE) represents isokinetic knee extension muscle strength expressed as a percentage of pre-exercise strength taken during the 14 days recovery.

Pre-exercise absolute values for isokinetic knee extension strength were 234 ± 18 Nm and 238 ± 9 Nm for CHO and WPH groups, respectively and were not significantly different. Univariate analysis revealed a significant main effect for time [F(3.6,43.2) = 21.897, P < 0.001, effect size(η^2^) = 0.646]. Similar to isometric strength, reductions in isokinetic knee extension strength (expressed as a percentage of pre-exercise strength) persisted for 7 days and were approximately 16% lower 24 hours post-exercise (P < 0.001), 20% (P < 0.001), 18% (P < 0.0001), and 11% (P < 0.01) lower 48 hours, 72 hours, and 96 hours into recovery, respectively, and 7% lower at day 7 (Figure 2). A moderate trend towards significance for group was identified [F(1,12) = 3.379, P = 0.091, effect size(η^2^) = 0.220], indicating that the reductions in strength also observed in the WPH group at the same time points of recovery were generally smaller than in the CHO group (Figure 2).

**Figure 3:**
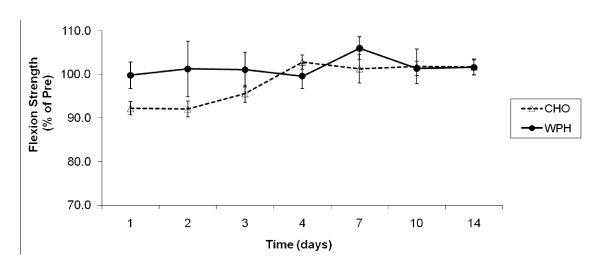
**Effect of CHO and WP on isokinetic knee flexion muscle strength after exercise-induced muscle damage**. Data (mean ± SE) represents isokinetic knee flexion muscle strength expressed as a percentage of pre-exercise strength taken during the 14 days recovery.

Pre-exercise absolute values for isokinetic knee flexion strength were 132 ± 8 Nm and 138 ± 5 Nm for CHO and WPH groups, respectively and were not significantly different. There was no significant main effect for time on the isokinetic knee flexion strength, indicating no significant change from pre-exercise strength values (Figure 3). A moderate trend towards significance for group main effect was observed [F(1,12) = 3.292, P = 0.095, effect size(η^2^) = 0.215]. This indicates that although minimal decrements in force were evident after the resistance exercises, the WPH group tended to have higher isokinetic knee flexion peak torque compared to the CHO group(Figure 3).

### Plasma Enzyme Activity

**Figure 4:**
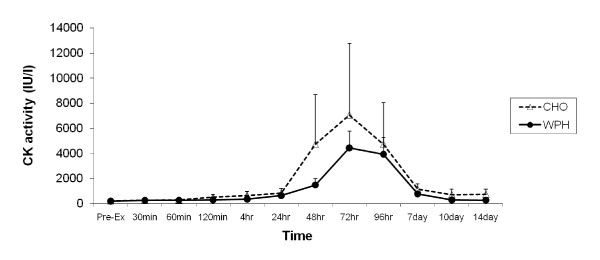
**Effect of CHO and WPH on plasma CK activity after exercise-induced muscle damage**. Data (mean ± SE) represents plasma CK activity (IU/l) taken during the 14 days recovery.

Pre-exercise CK levels were 225 ± 50 IU^.^1^-1 ^and 198 ± 50 IU^.^1^-1 ^in the CHO and WPH supplemented groups, respectively and were not significantly different. Univariate analysis revealed a significant time effect ([F(1,154) = 3.554, P < 0.001, effect size(η^2^) = 0.202) with no group or interactions detected. Figure 4. illustrates that CK activity was significantly elevated above baseline at 48 hours (P < 0.05), 72 hours (P < 0.05) and 96 hours (P < 0.05) post-exercise.

**Figure 5:**
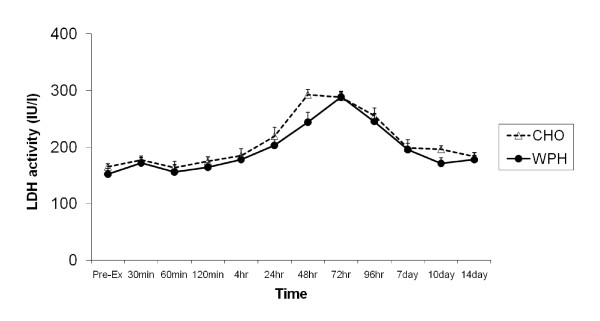
**Effect of CHO and WPH on plasma LDH activity after exercise-induced muscle damage**. Data (mean ± SE) represents plasma CK activity (IU/l) taken during the 14 days recovery.

Pre-exercise LDH levels were 155 ± 11 IU^.^1^-1 ^and 152 ± 10 IU^.^1^-1 ^in the CHO and WPH supplemented groups, respectively and were not significantly different. Univariate analysis revealed a significant time effect [F(11,121) = 23.937, P < 0.001, effect size(η^2^) = 0.685]. Figure 5. illustrates that LDH activity significantly changed over time being elevated above baseline at 24 hours (P < 0.0001), 48 hours (P < 0.0001), 72 hours (P < 0.0001), 96 hours (P < 0.0001) and at day 7 (p < 0.001) post-exercise. Similar elevations in plasma LDH activity were also observed in the WPH group. A trend towards significance for group [F(1,11) = 4.228, P = 0.064, effect size(η^2^) = 0.278] was also observed indicating LDH activity was generally lower in the WPH compared to CHO group throughout the recovery period.

## Discussion

The major finding of this study was that whey protein isolate supplementation resulted in an attenuation of the exercise-induced force reduction (isometric knee extension) compared to the carbohydrate control during the recovery period following exercise-induced muscle damage. A similar trend was also observed in isokinetic strength, with a further, tendency for lower LDH levels in the WPH group compared to the CHO group following the resistance exercise session. Most previous research into whey protein supplementation has examined its effects on muscle strength gains after resistance training. However, improved recovery from the acute bouts of exercises performed during the training sessions has been suggested as a possible mechanism for the beneficial effects observed in those studies [23]. The current study demonstrates that whey protein in a partially hydrolysed (pre-digested) form improves strength recovery rates, possibly due to an increase in the rate of repair processes and/or a reduction in the extent of damage, from intense training, in particular, eccentric exercise that is commonly used in weight training.

Following the eccentric contraction-based exercise session, isokinetic and isometric knee extension peak torque was significantly reduced and remained significantly lower than pre-exercise values for at least 4 days. In support of muscle damage producing these force decrements, plasma CK and LDH activity was increased during the days post resistance exercise, being significantly elevated above baseline 2 - 4 days into recovery. These observations were comparable to previous studies utilizing similar protocols to induce muscle damage [24-26].

In support of our hypothesis, WPH ingestion during recovery attenuated the decline in isometric extension strength compared to CHO group, with a similar trend in isokinetic knee extension. Interestingly, isokinetic knee flexion peak torque was not significantly affected by the resistance exercise session. This was primarily due to the very minimal decrements in muscle strength observed in the WPH group (close to 100% of pre-exercise values), such that the WPH group tended to have higher isokinetic knee flexion strength compared to the CHO group. Recent studies have confirmed that resistance exercise stimulates an increase in myofibrillar and sarcoplasmic proteins [27,28] as well as connective tissue proteins [29]. A single bout of resistance exercise results in the acute stimulation of muscle protein synthesis (up to 50-100% above basal values) that peaks within 3-24 hours, and can remain elevated, although at a diminishing rate, for up to 48 hours post-exercise [30-32]. Studies that have assessed both the rate of muscle protein breakdown and synthesis in response to a bout of resistance exercise have demonstrated that in a fasted state [31,32] the net muscle protein balance remains slightly negative. However, providing exogenous amino acids, especially within the first 4 hours after resistance exercise (as implemented in the present study), increases protein synthesis, decreases protein breakdown, and produces a positive protein balance [31,33], thus providing an environment for muscle growth. Although the aforementioned observations were not made with whey protein ingestion, a later study from the same laboratory confirmed the positive impact of whey protein supplementation on protein metabolism after resistance training exercise [34].

In the present study, oral ingestion of whey protein after the resistance exercise session most likely increased delivery of amino acids to the muscle, thus augmenting muscle protein synthesis and minimising protein degradation, thus producing the smaller reduction in force and/or faster recovery observed in the WPH group. Since neither muscle protein synthesis nor breakdown rates were measured, the relative balance cannot be determined. However, increased muscle protein synthesis is likely due to increased delivery of amino acids. Though not measured in the current study, recent results comparing protein fractionation on the bioavailability of amino acids clearly demonstrated significantly greater increases in the plasma concentrations of amino acids (and dipeptides) following protein hydrolysates compared to non-hydrolysed proteins [35],

Recent literature suggests that ingesting pre-digested proteins or free amino acids may be more advantageous during times of recovery from muscle damage compared to whole intact, slow absorbing proteins [12]. Indeed, Nosaka et al. [36], and more recently, White et al. [12] and Buckley et al. [13] clearly support this concept and findings observed in the current study. However, a limitation of the current study was the absence of another protein group (for example, whole intact protein such as milk) to make comparisons of this nature. Given the equivocal data on protein supplementation and muscle recovery, it can only be speculated that the beneficial effects of the protein source used in the current study was due to its hydrolysed, pre-digested form, and further research to clearly establish any difference is clearly warranted. Notwithstanding this, the positive protein balance created by increasing dietary intake of WPH following a single resistance exercise session would help to aid in recovery before subsequent exercise challenge during a resistance training program, thus allowing higher forces and hence training volumes to be achieved, eliciting greater strength benefits and muscle adaptations over time, as has been previously observed with WPH supplementation [23,37].

Whether WPH was also able to decrease the amount of damage produced by the eccentric exercise session is difficult to ascertain. Both groups exhibited increased CK and LDH loss from the muscle into the plasma, peaking 48 - 96 hours after exercise. The pattern of change in CK and LDH in the current study was similar to that following high force, eccentric exercise reported by [38]. However, plasma LDH levels were generally lower during recovery in the WPH group compared to the CHO group (P = 0.064), which may be indicative of less muscle fibre damage. Whey protein supplementation had no significant effect on plasma CK response after exercise which could be due to the extreme variability in CK response after exercise compared to the LDH response. Although CK is used as an indirect marker of muscle damage, there is a larger inter- and intra-participant variability in the CK response after exercise because blood concentrations reflect what is being released from damaged tissue as well as what is taken up by the reticuloendothelial system [39,40].

The beneficial effect from the whey protein supplement is likely due to its amino acid content, in particular the high essential amino acids (EAA) content, as opposed to any other constituents in the supplement. For instance, a carbohydrate drink with the same energy content as the protein supplement produces dramatic increases in blood glucose and insulin, but fails to stimulate protein synthesis [41,42]. Borsheim et al. [8] demonstrated that essential amino acids alone (without addition of carbohydrate) are an effective method for stimulating muscle protein synthesis following resistance training. Furthermore, in a later study by the same laboratory [43], adding 35 grams of carbohydrate to the amino acid mixture did not cause a greater stimulation of net muscle protein synthesis compared to the amino acids alone [43], showing that the stimulation of protein synthesis was clearly not a caloric effect of the supplement. Interestingly, since both groups were consuming the current recommended dietary allowance (RDA) for protein (0.8 g/kg/day) in sedentary individuals, the improvements in force recovery and reduced extent of damage can be attributed to the extra protein provided by the whey protein supplement.

However, increased protein synthesis is not likely to be the only contributing factor for the effects observed, particularly in the early stages of recovery. Nosaka et al. [36], showed that a mixture of amino acids was effective in reducing muscle soreness following eccentric exercise. A more recent study utilised only leucine, valine and isoleucine ingestion and observed the same effect 2-3 days following an eccentric exercise session [14], thus demonstrating the effectiveness of BCAA's in decreasing muscle soreness following exercise. Presumably, a maximal force effort would be more likely to be achieved if a person did not feel as much muscle soreness. Although Jackman et al. [14] did not observe improvements in muscle strength, perhaps the whey protein hydrolysate used in the present study not only supplied the BCAA's to reduce muscle soreness (although this was not measured), but also supplied all essential amino acids to maximise the increase in protein synthesis during recovery.

## Conclusion

In summary, the major finding of this investigation was that whey protein isolate supplementation elicited better maintenance of muscle strength in the days following contraction-induced eccentric muscle damage. This is likely due to increased protein synthesis due to the EAA contained within the WPH supplement, but could also be somewhat attributed to less damage to the muscle, as suggested by the trend for lower plasma LDH activity in the WPH group. Since the amino acid composition of whey proteins is very similar to that of skeletal muscle, whey protein supplementation may be providing amino acids essential for optimal muscle remodelling. Although the improvements elicited by whey protein supplementation appear small, an aggregation of those benefits with sustained, repeated training over time could still be of immense benefit for an athlete, providing even the smallest advantage, and may be the difference between winning and losing, or a faster return to competition. However, since untrained individuals were utilized in the current study (to ensure a robust damage response), any transferable benefits to the athletic population is speculative, although our previous research with recreational resistance-trained individuals does lend some support for this notion [10,22]. Future research should examine how different forms/fractions of proteins influence the rate of recovery and/or extent of damage following injury, and if training status plays an important role. Research into promoting functional recovery would not only have potential benefit for athletes, but could be of considerable benefit to a variety of populations, including those suffering from muscle wasting conditions, weakness associated with aging, neuromuscular disorders, acquired immunodeficiency syndrome, burn injury, cancer cachexia and prolonged sepsis.

## Competing interests

All researchers involved independently collected, analyzed, and interpreted the results from this study and have no financial interests concerning the outcome of this investigation.

## Authors' contributions

MC conceived the study, carried out the exercise sessions and all analyses, and drafted the manuscript. ER participated in the design of the study, helped with the enzyme analyses, and drafting of the manuscript. CS participated in the design of the study and the exercise sessions, and helped with the enzyme analyses and drafting of the manuscript. PC participated in the study design, participated in the exercise sessions and helped to draft the manuscript. AH helped conceive the study, participated in the study design and in the exercise sessions, helped with the strength measurements and helped to draft the manuscript. All authors read and approved the final manuscript.
